# SOCIAL SUPPORT BUFFERS THE EFFECT OF APOE4 GENE ON COGNITION

**DOI:** 10.1093/geroni/igad104.0324

**Published:** 2023-12-21

**Authors:** Ameante Payen, James Bateman, Jeanette Bennett

**Affiliations:** UNC Charlotte, Charlotte, North Carolina, United States; Wake Forest University School of Medicine, Winston-Salem, North Carolina, United States; University of North Carolina at Charlotte, Charlotte, North Carolina, United States

## Abstract

APOE4 allele is one of the most robust genetic risks for Alzheimer’s Disease (AD). It is associated with an earlier onset and conversion from Mild Cognitive Impairment (MCI) to AD dementia. Several studies have shown epigenetic interactions between the social context and the APOE4 allele’s relationship with cognition. Still, explorations of social risk factors for AD in genetically vulnerable populations are limited. This study explored whether the positive effects of social support (SS) on cognition are affected by APOE status in a sample of 115 older adults (n = 72.25, SD = 8.29). SS was measured using the Multidimensional Scale of Perceived Social Support (MSPSS), the preclinical Alzheimer’s Cognitive Composite-5 (PACC5) score to assess concurrent cognition, and the APOE polymorphism was determined via genotyping. As hypothesized, SS buffered the effect of APOE4 gene on PACC5 score, F(1, 107)=5.18, p = .02, Δ R2=.02. Specifically, older individuals who were positive for the allele displayed a significant and positive association between SS and mPACC5 (b=.19, p = .03). This relationship was not found for those who were APOE4 negative (b = -.04, p =.49). These results support the need to consider the epigenetic influence of social factors on later-life cognition, especially for those at risk for developing AD.

